# Prognostic impact of chronic lymphocytic leukemia comorbidity index in a young population: a real-world evidence study of a national gulf region cohort

**DOI:** 10.1186/s12885-024-12343-1

**Published:** 2024-05-13

**Authors:** Salem H. Alshemmari, Ahmad AlSarraf, Andy Kaempf, Alexey V. Danilov

**Affiliations:** 1 Department of Medicine, Faculty of Medicine, Kuwait University, State of Kuwait, PO BOX: 24923-23110 SAFAT, Jabriya, Kuwait; 2 Department of Hematology, Kuwait Cancer Center, Shuwaikh, Kuwait; 3 Biostatistics Shared Resource, Knight Cancer Institute, Oregon Health & Science University, Portland, OR USA; 4 Department of Hematology and Hematopoietic Stem Cell Transplant, City of Hope National Medical Center, Duarte, CA USA

**Keywords:** Chronic lymphocytic leukaemia, Comorbidities, Progression, Prognosis, Biology

## Abstract

In chronic lymphocytic leukaemia (CLL), comorbidities assessed by the CLL comorbidity index (CLL-CI) have been associated with outcomes in Western cohorts. We conducted a retrospective analysis of an unselected Middle Eastern cohort of newly diagnosed CLL patients seen at the Kuwait Cancer Control Center (*n* = 300). Compared to Western studies, these Middle Eastern patients were diagnosed at a younger age (median of 59) and had a higher comorbidity burden (69% non-low risk CLL-CI). A higher CLL-CI score was independently associated with significantly shorter event-free survival and greater risk of death. Our analysis demonstrates that CLL-CI is a valuable tool for comorbidity assessment and prognostic influence in (relatively young) Middle Eastern CLL patients.

## Introduction

Chronic lymphocytic leukemia (CLL) is a hematological malignancy characterized by an indolent course that, in some cases, can progress and lead to death [1]. To predict progression potential, several prognostic scores have been developed, incorporating both biological and clinical factors [2, 3].

Comorbidities are frequently present at CLL diagnosis and have been found to influence prognosis [4]. However, they are not taken into account in commonly-used prognostic scores such as the CLL-IPI [2, 5]. The cumulative illness rating scale (CIRS) has been utilised as a predictive tool and as inclusion criteria for clinical trials [6, 7]. However, CIRS requires an assessment of 14 different organ systems, which is clinically impractical. The recently proposed CLL-comorbidity index (CLL-CI) is a CIRS-based prognostic index of reduced complexity that only evaluates upper gastrointestinal (GI), endocrine, and vascular comorbidities, as these were most strongly associated with event-free survival (EFS) in a 10-institution U.S. cohort of CLL patients [8]. A national Danish CLL registry study then confirmed the ability of CLL-CI to predict time to first treatment (TTFT), EFS, and overall survival (OS), with the latter two outcomes measured from both diagnosis and first treatment [9]. The Middle Eastern CLL population, however, is much younger (median age at CLL diagnosis of 59 years) than in the West (median age of 70 years) and has a different comorbidity profile [10, 11].

The aim of this study was to assess CLL patient comorbidities using CIRS in order to evaluate the prognostic validity of the CLL-CI in Middle Eastern patients receiving care at the Kuwait Cancer Control Center (KCCC), the only cancer center in Kuwait.

## Methodology

### Patients

Starting from 322 consecutive CLL patients seen at KCCC over the past 25 years, our analysis population included the 300 patients with recorded data for birth and diagnosis date, disease stage at diagnosis, treatment start date (if known to have received CLL therapy), post-diagnosis follow-up, and sufficient comorbidity information to compute CLL-CI. Patient and disease characteristics obtained at or near diagnosis included age, Binet stage, *IGHV* and *TP53* mutational status, and cytogenetic abnormalities via FISH. Patient comorbidities determined at the KCCC around the time of diagnosis were used to calculate the CLL-CI score (1 point apiece for any vascular, at least moderate upper GI, and at least moderate endocrine comorbidity). CLL-CI risk groups were defined – per the original U.S. study – as “low” (0 points), “intermediate” (1 point), and “high” (2–3 points). The study was approved by the institutional review board (2017/523) and all patients enrolled in the study provided written informed consent.

### Statistical analysis

Baseline characteristics of our newly diagnosed CLL Middle Eastern cohort were summarized with frequencies and medians with interquartile ranges (IQR) and compared across the three CLL-CI risk groups by Fisher’s exact test or Kruskal-Wallis test depending on if the characteristic was categorical or continuous, respectively. Median follow-up was estimated using the reverse Kaplan-Meier approach. EFS and OS were each measured from CLL diagnosis and EFS events included treatment initiation, disease progression, and any-cause death. Survival distributions were estimated by the Kaplan-Meier method and log-rank tests were conducted to assess differences in EFS and OS across CLL-CI groups. Cox regression models were fit to both survival outcomes to estimate unadjusted (in univariable models) and adjusted (in multivariable models) hazard ratios (HRs) and Wald test p-values. The significance of the overall effect of CLL-CI (a 3-group factor) on survival was determined by a 2 degree of freedom Wald test using Cox model estimates. Cox models that included *TP53* as a predictor utilized left truncation to account for the variable time between diagnosis and ascertainment of *TP53* status (via FISH and/or mutational testing) as these tests were not always performed at diagnosis. However, only *TP53* information that was acquired within 6 months of diagnosis and before an EFS event was included. Harrell’s concordance index (c-index) was estimated to assess Cox model discrimination. P-values < 0.05 were considered statistically significant. R version 4.2.1 (R Core Team, 2022) was used to perform statistical analyses and create figures.

## Results

The patient characteristics of our study cohort are provided in Table 1. The median age was 59 years (IQR, 52–68), male to female ratio was 3:1, and 90% were of Middle Eastern (ME) descent (9% had South Asian and 1% had other descent). Comparatively, the median age at CLL diagnosis was 71 years in the Danish population-based cohort and the median age at treatment was 67 years in the multi-center U.S. study defining CLL-CI [8, 9]. At diagnosis, 222 patients (74%) presented with Binet stage A disease whereas 39 (13%) and 39 (13%) had Binet stage B and C, respectively. Among the 165 patients (55%) with evaluable FISH and/or *TP53* mutation testing within 6 months of diagnosis, 8 (5%) had a *TP53* aberration. The majority of patients (*n* = 194; 65%) underwent testing for *IGHV* mutational status, with 56% of these having unmutated *IGHV*.

**Table 1:** Patient characteristics stratified by CLL-CI group

Patient feature	Low risk (*n* = 93; 31%)	Intermed. risk (*n* = 137; 46%)	High risk (*n* = 70; 23%)		Overall (*n* = 300)	*p*-value
**Age (y)**						< 0.001
Median (IQR)	55.9 (49.1–63.8)	59.2 (53.0-66.2)	66.3 (58.4–74.4)		59.3 (52.4–67.6)	
**Sex**						0.020
Male (%)	79 (84.9)	96 (70.1)	49 (70.0)		224 (74.7)	
Female (%)	14 (15.1)	41 (29.9)	21 (30.0)		76 (25.3)	
**Ethnic descent**						0.644
ME (%)	81 (87.1)	123 (89.8)	66 (94.3)		270 (90.0)	
S. Asian (%)	10 (10.8)	12 (8.8)	4 (5.7)		26 (8.7)	
Other (%)	2 (2.2)	2 (1.5)	0 (0.0)		4 (1.3)	
**Binet stage**						0.833
A (%)	71 (76.3)	99 (72.3)	52 (74.3)		222 (74.0)	
B (%)	9 (9.7)	20 (14.6)	10 (14.3)		39 (13.0)	
C (%)	13 (14.0)	18 (13.1)	8 (11.4)		39 (13.0)	
**Upper GI disease** ^a^						NA
Yes (%)	0 (0)	43 (31.4)	44 (62.9)		87 (29.0)	
No (%)	93 (100)	94 (68.6)	26 (37.1)		213 (71.0)	
**Endocrine disease** ^a^						NA
Yes (%)	0 (0)	84 (61.3)	66 (94.3)		150 (50.0)	
No (%)	93 (100)	53 (38.7)	4 (5.7)		150 (50.0)	
**Vascular disease** ^b^						NA
Yes (%)	0 (0)	10 (7.3)	39 (55.7)		49 (16.3)	
No (%)	93 (100)	127 (92.7)	31 (44.3)		251 (83.7)	
*IGHV*status^*^; *n* = 194						0.092
Mutated (%)	30 (55.6)	39 (43.3)	17 (34.0)		86 (44.3)	
Unmutated (%)	24 (44.4)	51 (56.7)	33 (66.0)		108 (55.7)	
Del(17p) or*TP53*mutation^*^; *n* = 165						0.019
Yes (%)	0 (0)	3 (4.0)	5 (12.5)		8 (4.8)	
No (%)	50 (100)	72 (96.0)	35 (87.5)		157 (95.2)	

^a^ CIRS score of at least 2. ^b^ CIRS score of at least 1. NA = no test performed across CLL-CI groups when the patient feature is a CLL-CI component. ^*^The total does not add up to 300 owing to missing data that were either unavailable, not close enough to CLL diagnosis to be included in a prognostic model, or not obtained before a patient was treated or had disease progression (as the primary outcome was post-diagnosis EFS). P-values are from Fisher exact tests for categorical variables and the Kruskal–Wallis test for patient age at diagnosis

Assessment of the CLL-CI-quantified comorbidity burden at diagnosis showed that 93 patients (31%) were low risk, 137 (46%) intermediate risk, and 70 (23%) high risk. Regarding the CIRS components that comprise CLL-CI, 150 (50%) of our patients had an endocrine comorbidity of at least moderate grade, 87 (29%) had a moderate or severe upper GI condition, and 49 (16%) had at least mild vascular comorbidities. For endocrine-metabolic comorbidities qualifying as moderate or severe by CIRS, 41% of KCCC patients had diabetes treated with oral agents or insulin, 10% had dyslipidaemia requiring medication, and 11% had other endocrine-related problems (e.g., thyroid disorders). The percentages of patients in our Middle Eastern cohort with other CIRS body system comorbidities (of mild, moderate, or severe grade) at CLL diagnosis were: 44% with hypertension, 9% with non-renal genitourinary, and 8% with musculoskeletal. The median total CIRS score was 5 (IQR, 2–8; range, 0–20). This is likely an underestimate as while medical comorbidities which impact disease management are well documented in our medical records, certain conditions (e.g., poor vision or hearing) are not routinely recorded, making accurate quantification of total CIRS challenging in retrospective analyses.

**Fig. 1:**
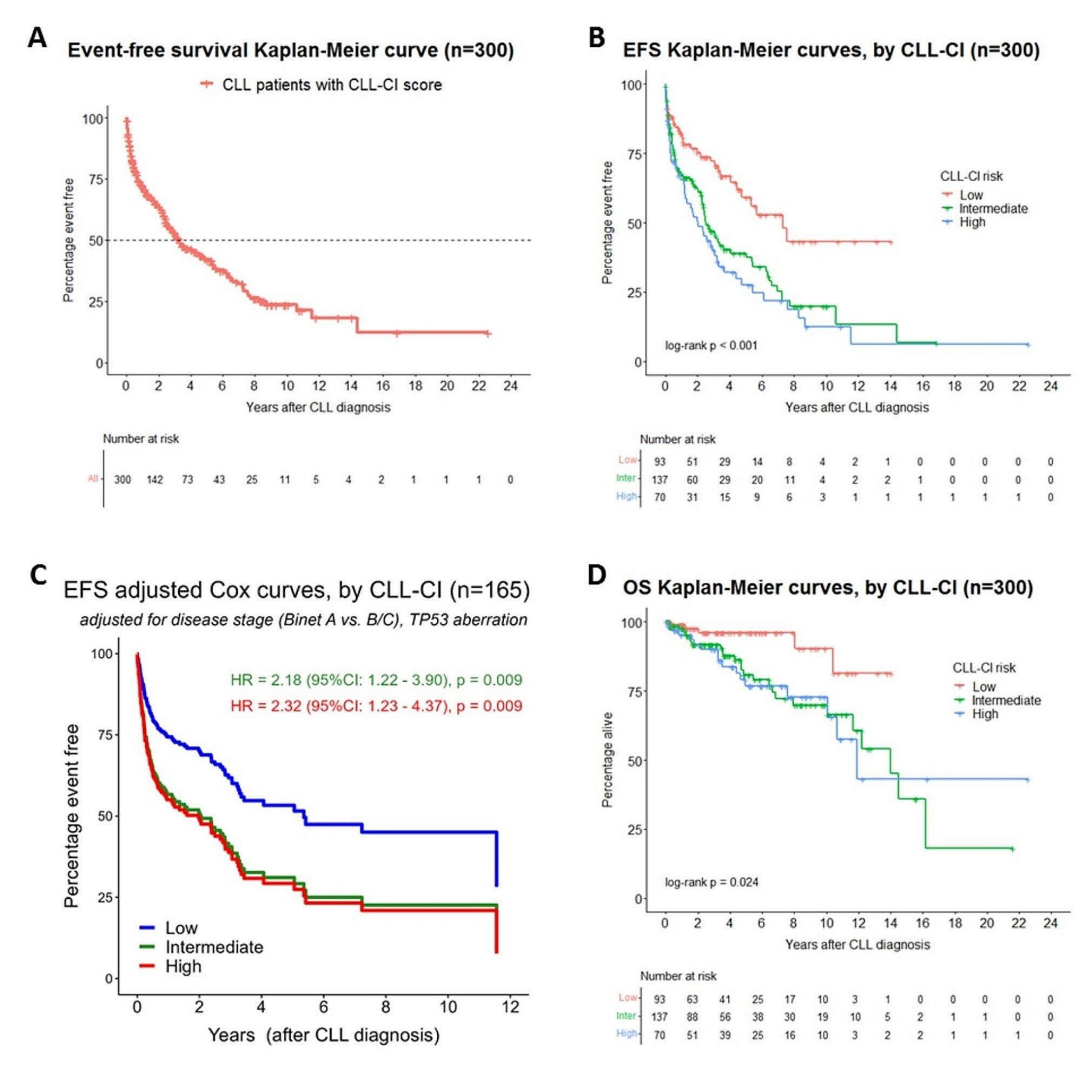
Event-free and overall survival curves. Event-free survival Kaplan-Meier curves of the entire cohort **(A)** and by CLL-CI risk group **(B)**. EFS curves for each CLL-CI group adjusted for disease stage and *TP53* aberration (via creation of an artificial dataset with balanced CLL-CI distribution) among the subset of patients with known *TP53* status within 6 months of diagnosis **(C)**. Overall survival Kaplan-Meier curves for each CLL-CI group **(D)**

Median post-diagnosis follow-up was 49 months (95% confidence interval [CI], 42–61), 48 patients died (16%), and 139 (46%) received treatment for CLL, with treatment initiated at a median of 11 months (IQR, 3–36) after diagnosis. 54% (163 patients) had an EFS event during follow-up and the median EFS was 39 months (95%CI, 30–57) (Fig. 1A). Stratifying by CLL-CI revealed a statistically significant difference in EFS (log-rank *p* < 0.001), with median EFS of 88 (95%CI, 53-NA), 30 (95%CI, 27–48), and 24 (95%CI, 14–39) months in the low, intermediate, and high risk groups, respectively (Fig. 1B).

Evaluating the association between individual CLL-CI components and EFS in our Middle Eastern cohort revealed that neither presence of moderate/severe endocrine comorbidities (HR = 1.24, *p* = 0.164) nor vascular conditions (HR = 1.05, *p* = 0.828) were significant predictors of EFS (Table 2). However, moderate or severe comorbidity involving the upper GI system was strongly associated with a shorter EFS in both a univariable (*n* = 300; HR = 2.05, *p* < 0.001) and a multivariable Cox model with disease stage and *TP53* status as covariates (*n* = 165; HR = 2.36 [95%CI, 1.51–3.68], *p* < 0.001; c-index = 0.73).

**Table 2:** Univariable Cox regression analysis of EFS and OS measured from CLL diagnosis

Predictor	Category	Count (%)	EFS univariable Cox: HR (95% CI); *p*-value	OS univariable Cox: HR (95% CI); *p*-value
Age at diagnosis	< 65^^^ ≥ 65	202 (67.3%) 98 (32.7%)	1.21 (0.87–1.68); 0.263	4.17 (2.33–7.45); <0.001
Sex	female^^^ male	76 (25.3%) 224 (74.7%)	0.83 (0.59–1.18); 0.307	0.54 (0.30–0.95); 0.034
Ethnic descent	Middle East^ South Asian Other	270 (90.0%) 26 (8.7%) 4 (1.3%)	0.98 (0.56–1.73); 0.946 0.84 (0.21–3.40); 0.807	0.25 (0.03–1.84); 0.174 Unstable estimate
Advanced stage (i.e., Binet B/C)	no^^^ yes	222 (74.0%) 78 (26.0%)	4.25 (3.10–5.83); <0.001^†^	1.90 (1.05–3.42); 0.032
*IGHV* status	mutated^^^ unmutated unknown	86 (28.7%) 108 (36.0%) 106 (35.3%)	4.15 (2.58–6.67); <0.001 N/A	19.00 (2.54–141.9); 0.004
Del(17p) or *TP53* mutation^¥^	no^^^ yes unknown	157 (52.3%) 8 (2.7%) 135 (45.0%)	4.64 (1.98–10.8); <0.001 N/A	15.54 (3.68–65.7); <0.001^†^
Vascular comorb. with CIRS ≥ 1	no^^^ yes	251 (83.7%) 49 (16.3%)	1.05 (0.70–1.55); 0.828	1.96 (1.03–3.71); 0.039^†^
Upper GI comorb. with CIRS ≥ 2	no^^^ yes	213 (71.0%) 87 (29.0%)	2.05 (1.50–2.80); <0.001	0.48 (0.24–0.98); 0.045
Endocrine comorb. with CIRS ≥ 2	no^^^ yes	150 (50.0%) 150 (50.0%)	1.24 (0.91–1.69); 0.164	3.18 (1.62–6.24); 0.001
CLL-CI risk	low^^^ intermed. high	93 (31.0%) 137 (45.7%) 70 (23.3%)	1.98 (1.32–2.97); 0.001 2.43 (1.56–3.77); <0.001	3.33 (1.28–8.68); 0.014 3.49 (1.27–9.56); 0.015

^ = reference level for Hazard Ratios. † = Cox model proportional hazards assumption violated. ¥*TP53* status – presence vs. absence of del(17p) via FISH or mutation via sequencing – must have been ascertained within 6 months of diagnosis and before first treatment and progression to be recorded in this table (because patient diagnosis date is the start time for survival analyses); values ascertained outside this time window are shown as “unknown”. Note that patients with “unknown” values for a specific predictor were not included in the Cox models

Multivariable Cox regression applied to the 165 patients with evaluable *TP53* status revealed that CLL-CI remained a significant predictor of EFS (intermediate vs. low risk: HR = 2.18 [95%CI, 1.22–3.90], *p* = 0.009; high vs. low risk: HR = 2.32 [95% CI, 1.23–4.37], *p* = 0.009; c-index = 0.73) after adjusting for disease stage (Binet A vs. Binet B/C) and the presence of del(17p) or *TP53* mutation (Fig. 1C). As *TP53* disruption was rare (5% of tested patients) and unknown in 45% of patients, we also examined the independent effect of CLL-CI in the 300-patient full cohort when adjusting for disease stage, the only non-CIRS feature significantly related to EFS and recorded in all patients (Table 2). We also separately analysed the 194 patients with known *IGHV* status where we adjusted for disease stage and *IGHV*. In these multivariable models, higher CLL-CI risk was a significant predictor of shorter EFS in both the full cohort (intermediate vs. low risk: HR = 1.79 [95%CI, 1.19–2.69], *p* = 0.005; high vs. low risk: HR = 2.55 [95% CI, 1.64–3.96], *p* < 0.001; c-index = 0.73) and the *IGHV*-known cohort (intermediate vs. low risk: HR = 1.74 [95%CI, 0.98–3.08], *p* = 0.057; high vs. low risk: HR = 2.38 [95% CI, 1.28–4.42], *p* = 0.006; c-index = 0.79).

CLL-CI was significantly associated with OS (log-rank *p* = 0.024) in the absence of covariates. Although the OS curve for low risk CLL-CI patients (median not reached) was well-segregated, there was minimal separation between curves for intermediate risk (median of 168 months) and high risk (median of 143 months) patients (Fig. 1D). When adjusting for age ≥ 65 at diagnosis (which was a significant predictor of OS in the univariable model, see Table 2), disease stage, and *TP53* aberration, intermediate/high risk CLL-CI patients had shorter OS at borderline significance levels (*n* = 165; intermediate vs. low risk: HR = 4.59 [95%CI, 1.00-20.96], *p* = 0.049; high vs. low risk: HR = 3.99 [95%CI, 0.85–18.68], *p* = 0.079; c-index = 0.70). However, CLL-CI did not correlate with OS (intermediate vs. low risk: HR = 1.47, *p* = 0.532; high vs. low risk: HR = 1.17, *p* = 0.819) when adjusting for *IGHV* status (along with age ≥ 65 and disease stage) in the 194 evaluable patients with 23 observed deaths.

Interestingly, in contrast to our EFS findings, both moderate/severe endocrine comorbidities (HR = 3.18, *p* = 0.001) and any vascular condition (HR = 1.96, *p* = 0.039) were significant predictors of OS in the univariable setting. The presence of a vascular comorbidity was also an independent predictor of OS upon adjusting for other prognostic factors (HR = 2.49 [95%CI, 1.05–5.90], *p* = 0.038; c-index = 0.73). Furthermore, although moderate/severe upper GI comorbidity was an independent significant predictor of shorter EFS, it does not have a negative impact on OS (HR = 0.71 [95%CI, 0.29–1.72], *p* = 0.446; c-index = 0.65) and is thus inferior to CLL-CI in terms of predicting clinical outcome from the time of diagnosis.

## Discussion

In contrast to Western countries, the Middle Eastern population tends to be younger, potentially with a different profile of comorbidities [10, 11]. Comorbidities have been shown to impact survival outcomes in patients with CLL [4]. Therefore, it is crucial to have a clinically applicable and validated tool for assessing comorbidities in populations with distinct demographics, disease etiologies, and prevalent comorbid conditions. As our findings indicate that newly diagnosed CLL patients from the Gulf region seen at KCCC are, on average, about 10 years younger than their Western counterparts, there is an unmet need to validate any CLL prognostic index in this Middle Eastern population.

Despite the younger age of Middle Eastern patients with CLL, our cohort exhibited a higher burden of comorbidities compared to Western cohorts. In the aforementioned U.S. and Danish cohorts, 42% and 63% of patients, respectively, were classified as low risk according to the CLL-CI, whereas 31% of our cohort met this criteria [8, 9]. Conversely, the proportion of high risk patients in our cohort is also higher, at 23% compared to 20% and 7% in the U.S. and Danish cohorts, respectively. This might be due to referral bias, since KCCC is a tertiary care facility where high risk cases may be overrepresented compared to uncomplicated indolent cases that are monitored in community clinics.

Our data show that the Middle Eastern population had a distinct comorbidity profile compared to the West. Musculoskeletal and vascular comorbidities, which include coronary artery and cerebrovascular diseases, were less common in our CLL population than in an older Western population [4, 8, 9, 12]. In contrast, the prevalence of hypertension in our CLL cohort was comparable to that in the U.S. study. Diabetes mellitus requiring treatment was relatively more common in CLL patients seen at KCCC, which might result from the high prevalence of obesity in the Arabian Gulf region in general and in Kuwait in particular [13, 14]. Metabolic syndrome may be involved in the underlying pathogenesis of CLL in such patients because it is implicated in carcinogenesis mediated by chronic inflammation [15]. Whether this factor contributes to CLL onset at a younger age in the Gulf region remains to be determined. Finally, moderate-to-severe upper GI comorbidities, primarily the use of proton pump inhibitors (PPI), were more common in our CLL cohort than in the Danish cohort, possibly related to obesity and metabolic syndrome.

Upper GI comorbidities and the use of PPIs are associated with disruption of the gut microbiome [16]. Moreover, reduced gut microbial diversity has been observed in patients with CLL and is thought to contribute to disease development and/or be a manifestation of the underlying immune dysfunction [17]. Alterations in the gut microbiome are also implicated in the development, progression, and response to treatment of other cancer types [18].

In our young population, the CLL-CI was an independent, significant predictor of EFS even after adjusting for biological and other clinical parameters. However, there was no prognostic distinction between the intermediate and high risk groups. Similarly, the U.S.-based CLL-CI study showed the greatest prognostic distinction between low risk and intermediate risk groups [8]. Although intermediate or high risk CLL-CI was associated with shorter OS, this effect disappeared when *IGHV* status was controlled for; yet it is worth noting that *IGHV* was only available in 65% of patients. A large prospective study revealed that comorbidities contribute to non-CLL-specific death (i.e., death resulting from the comorbidity itself) rather than CLL-specific death (i.e., death secondary to disease progression or infection) [12]. This might, in part, explain why this comorbidity index was a stronger predictor of post-diagnosis CLL progression (captured by EFS) than of any-cause death in our cohort. Moreover, given that our patient cohort was relatively young (median age of 59) and early stage (74% Binet A), longer follow-up may be needed to better discern the impact of CLL-CI on OS in Middle Eastern patients.

The presence of *del(17p)* or *TP53* mutation, although observed in only 5% of evaluable patients in our cohort, emerged as a significant predictor of outcome. This highlights the prognostic relevance of these genetic aberrations, underscoring the importance of molecular markers such as *TP53* mutation status in decision-making for CLL patients even in the era of targeted agents [19].

Our study is not without limitations. This validation of CLL-CI is based on a modest sample size (300 patients, 165 with peri-diagnostic *TP53* information) and we did not evaluate the prognostic impact of total CIRS score due to the challenge of capturing all CIRS components upon retrospective review. Additionally, the relatively short follow-up period (∼ 50 months), low proportion of deaths (16% of patients), and lack of cause of death information (to parse out non-CLL-specific deaths) may have hindered the index’s ability to predict OS. Nevertheless, in demonstrating that CLL-CI is an independent, significant predictor of time to first progression, this index seems capable of informing upfront decisions about patient management and treatment.

In conclusion, the CLL-CI is an acceptable clinical tool for comorbidity assessment that is associated with adverse outcomes in younger patients with CLL. Practicing physicians might find it applicable in day-to-day practice. Furthermore, it can be used to guide clinical trial design. Further studies are needed to evaluate the CLL-CI’s performance alongside other commonly used prognostic indices in the young Middle Eastern population diagnosed with CLL.

## Data Availability

The datasets used and/or analysed during thecurrent study available from the corresponding author on reasonable request.
